# Convergent Evolution in SARS-CoV-2 Spike Creates a Variant Soup from Which New COVID-19 Waves Emerge

**DOI:** 10.3390/ijms24032264

**Published:** 2023-01-23

**Authors:** Daniele Focosi, Rodrigo Quiroga, Scott McConnell, Marc C. Johnson, Arturo Casadevall

**Affiliations:** 1 North-Western Tuscany Blood Bank, Pisa University Hospital, 56124 Pisa, Italy; 2 Instituto de Investigaciones en Físico-Química de Córdoba (INFIQC-CONICET), Facultad de Ciencias Químicas, Universidad Nacional de Córdoba, Cordova 5000, Argentina; 3 Department of Molecular Microbiology and Immunology, Johns Hopkins Bloomberg School of Public Health, Baltimore, MD 21205, USA; 4 Department of Molecular Microbiology and Immunology, University of Missouri School of Medicine, Columbia, MO 65201, USA

**Keywords:** SARS-CoV-2, Spike, omicron, convergent evolution, R346, K444, BQ.1.1, XBB, Evusheld™, cilgavimab, tixagevimab, bebtelovimab

## Abstract

The first 2 years of the COVID-19 pandemic were mainly characterized by recurrent mutations of SARS-CoV-2 Spike protein at residues K417, L452, E484, N501 and P681 emerging independently across different variants of concern (Alpha, Beta, Gamma, and Delta). Such homoplasy is a marker of convergent evolution. Since Spring 2022 and the third year of the pandemic, with the advent of Omicron and its sublineages, convergent evolution has led to the observation of different lineages acquiring an additional group of mutations at different amino acid residues, namely R346, K444, N450, N460, F486, F490, Q493, and S494. Mutations at these residues have become increasingly prevalent during Summer and Autumn 2022, with combinations showing increased fitness. The most likely reason for this convergence is the selective pressure exerted by previous infection- or vaccine-elicited immunity. Such accelerated evolution has caused failure of all anti-Spike monoclonal antibodies, including bebtelovimab and cilgavimab. While we are learning how fast coronaviruses can mutate and recombine, we should reconsider opportunities for economically sustainable escape-proof combination therapies, and refocus antibody-mediated therapeutic efforts on polyclonal preparations that are less likely to allow for viral immune escape.

## 1. Introduction

In the third year of the COVID-19 pandemic, a relevant proportion of the general population is now largely protected from severe COVID-19 disease and death by mass vaccination campaigns and by immunity from former infection, as shown by the decongestion of hospitals in the western hemisphere. Unfortunately, SARS-CoV-2 remains a life-threatening pathogen for frail and immunocompromised (IC) patients who are unable to mount a protective immune response [1]. IC individuals create a cohort population in whom the virus can persistently replicate, which is a novelty for pandemics [2]. In this regard, advancements in therapeutics and supportive care have increased the prevalence of IC patients up to 2.8% compared to just a few decades ago [3]. SARS-CoV-2 infection in IC patients is arguably the most difficult current problem in the COVID-19 pandemic: these individuals can have large viral loads which inevitably include antigenically different viruses and have a diminished capacity for clearing the infection [2].

**Figure 1:**
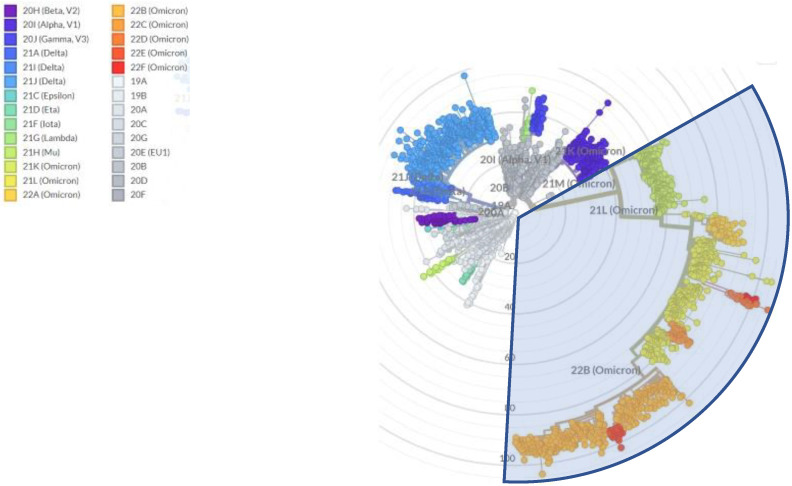
Radial tree of SARS-CoV-2 evolution, with branch length approximating divergence, showing that Omicron (light blue shadow) currently includes more than 45% or variations across 3193 genomes sampled between December 2019 and December 2022. Accessed online at https://nextstrain.org/ncov/gisaid/global/all-time?l=radial&m=div (accessed on 26 December 2022).

Since Summer 2022, SARS-CoV-2 transmission has proceeded undisturbed worldwide after the relaxation of nonpharmaceutical interventions such as lockdowns, social distancing, and face masks, which together with the waning of infection- and vaccine-elicited immunity, has increased opportunities for spread and the number of susceptible individuals, respectively. Hence, the increase in the “human culture medium” has led to large infectious waves during 2022, with estimated excess deaths similar to those observed in 2020 [4]. While acquisition and waning of immunity from former infections is not a novel occurrenece, in the COVID-19 pandemic the timely introduction of vaccination campaigns and therapeutics targeting the viral receptor domain has has the potential to alter the course of a coronavirus pandemic. There is no historical precedent for the current situation. The combined action of increasing cumulative viral loads in the “human culture medium” and such selective pressures has led to an unprecedented increase in viral diversification in 2022. WHO nomenclature for variants of concern remained stuck at “Omicron” [5], while alternative naming schemes introduced novel names to designate lineages that are responsible for thousands of hospitalizations. The most refined phylogeny to date has been released by PANGOLIN which counts more than 650 designated Omicron sublineages at the time of writing (https://github.com/cov-lineages/pango-designation) (accessed on 26 December 2022)), accounting for more than 45% of SARS-CoV-2 variability (Figure 1). Of interest, such increase in divergence was detected despite a 75% reduction in genomic surveillance in 2022. After peaking at 1 million sequences in January 2022, the number of new sequences deposited at the site decreased to 248,000 in November 2022 (https://cov-spectrum.org/explore/World/AllSamples/Past6M/sequencing-coverage (accessed on 26 December 2022)). Consequently, it is likely that the number of defined circulating sublineages is an underestimate of the viral genetic variation in the current pandemic.

## 2. Mutation Rates and Mutational Spectra

Mutation rate (MR) is often used interchangeably to indicate 2 different things: occurrence of mutations within a single host (intrahost evolution at individual level without any demand for outcompeting co-circulating strains) or step-wise accumulation of mutations (“antigenic drift”) that get fixed within a species. While the first meaning has been demonstrated (e.g., in IC hosts [6,7,8], and after administration of the small molecule antiviral molnupiravir which known to increase G→A and C→U transition mutations [9]), from an evolutionary standpoint it is the second meaning which is more interesting and already well-established for other respiratory pathogens [10], including the related human coronavirus 229E [11].

Early in the pandemic, data suggested that mass vaccination could restrict SARS-CoV-2 mutation rates (MR): the diversity of the SARS-CoV-2 lineages declined at the country-level with increased rate of mass vaccination (*r* = −0.72) and vaccine breakthrough patients harbor viruses with 2.3-fold lower diversity in known B cell epitopes compared to unvaccinated COVID-19 patients [12]. Additionally, vaccination coverage rate was inversely correlated to the MR of the SARS-CoV-2 Delta variant in 16 countries (*r*^2^ = 0.878) [13].

Ruis et al. found a halving in the relative rate of G→T mutations in Omicron compared to pre-Omicron sublineages [14]. To exclude selective pressures on the derived protein structures, Bloom et al. found similar results by repeating the analysis focusing on 4-fold degenerate codons (i.e., codons that can tolerate any point mutation at the third position, although codon usage bias restricts this in practice in many organisms) [15]. Replicaion of viruses and bacteria in the lower respiratory tract has been associated with high levels of G > T mutations and for SARS-CoV-2 this effect occurred with Delta but was lost in Omicron [14]. Such changes on mutation type and rate could theoretically stem from from mutations affecting genome replication and packaging [16], as well as from mutations in genes encoding proteins that antagonize host innate-immune factors (e.g., APOBEC), which otherwise will mutate viral nucleic acids [17,18,19] and/or from environmental factors [9].

**Figure 2:**
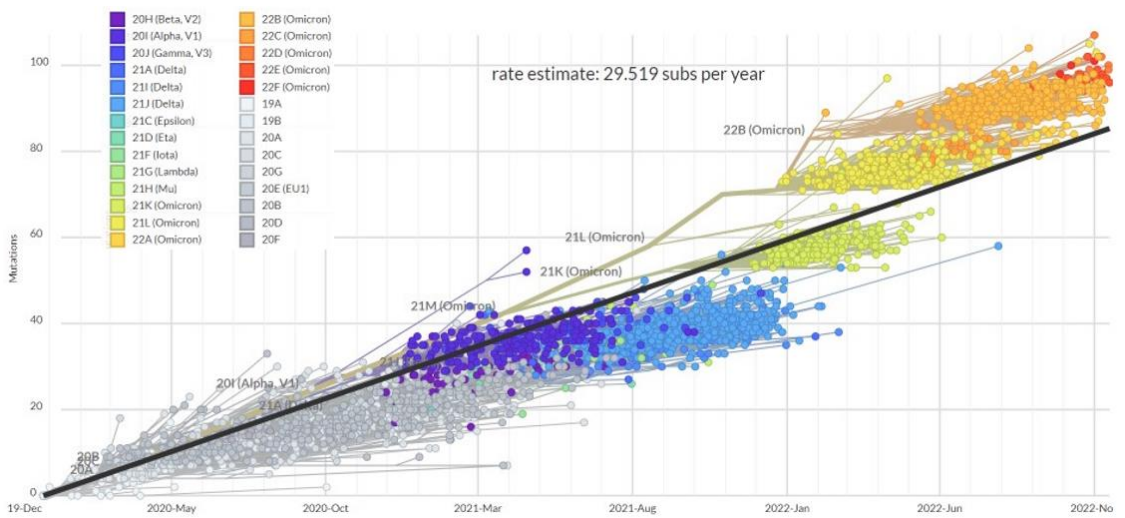
Clock tree of SARS-CoV-2 evolution, with regression line showing an increase in the estimate rate of substitutions per year across 3045 genomes sampled between December 2019 and November 2022. Accessed online at https://nextstrain.org/ncov/gisaid/global/all-time?l=clock&m=div (accessed on 26 November 2022).

The average MR of the entire SARS-CoV-2 genome was estimated from the related mouse hepatitis virus (MHV) to be 10^−6^ nucleotides per cycle, or 4.83 × 10^−4^ subs/site/year, which is similar, or slightly lower, that observed for other RNA viruses [20]. Following the removal of mandatory nonpharmaceutical interventions such as face masks, social distancing, and quarantine in most western countries, vaccination was not sufficient to prevent hyperendemicity. The MR of SARS-CoV-2 consequently doubled from 23 substitutions per year before December 2021 to 45 substitutions per year after December 2021, coinciding with the advent of omicron (Figure 2), which approximates 14.5/subs/year for the ~30 kb SARS-CoV-2 genome. This rate should set the upper limit for mutation frequency, as many mutations will not be viable and/or transmissible and thus not observed in the sequencing data at baseline. Despite this, the previously acknowledged reductio in sequencing intensity in 2022 leaves some room for higher MR. It had been previously shown that the P203L mutation in the error-correcting exonuclease non-structural protein 14 (nsp14) almost doubles the genomic MR (from 20 to 36 SNPs/year) [21]. While this change is not prevalent in Omicron lineages, many changes in the replication machinery appeared with Omicron, such as K38R, Δ1265, and A1892T in Nsp3; P132H in Nsp5; I189V in Nsp6; P323L in Nsp12; and I42V in Nsp14, and some of them could have contributed to the MR jump [22].

## 3. Convergent Evolution

In the midst of such massive lineage divergence, convergent evolution towards certain motifs has become increasingly manifest.

In the pre-Omicron and pre-vaccine era, variants of concern (VOCs) notably converged to mutations which resulted in the following amino acid changes: K417N (Beta and Gamma), L452R (Delta), E484K (Beta and Gamma), N501Y (Alpha, Beta, Gamma) and P681X (Alpha and Delta) [23]. These amino acid changes have been proposed to increase the stability of the trimeric protein [24,25,26], and they emerged in the absence of significant selective pressures by the immune system. K417N, E484A, N501Y and P681H remained hallmarks of BA.2.*, while the BA.2-paraphyletic BA.4/5 (i.e., a clade stemming from BA.2) acquired L452R and F486V and the Q493R reversion.

**Figure 3:**
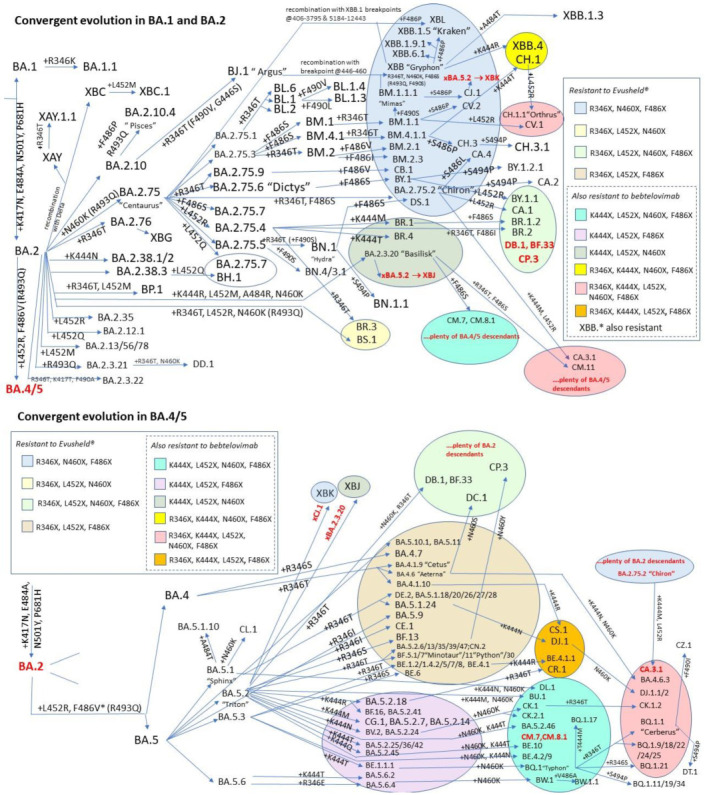
Diagram representing all SARS-CoV-2 Omicron sublineages designated by PANGOLIN as of 26 December 2022 for which at least one of the Spike RBD immune escaping mutations (R346X, K444X, L452X, N460X, F486X, or R493Q) represents a branching event. Mythological names introduced by T Ryan Gregory and used colloquially are also reported. Convergence towards combos of this mutations is noted, with different background colors representing different combinations. Resistance of each combination to clinically authorized anti-Spike mAbs is reported in the squared box. For visualization purposes, the upper panel shows BA.1 and BA.2 evolution, while the lower panel shows BA.4/5 evolution.

In the last year the BA.2 variant first generated a wave that led first to the paraphyletic BA.4/5 sublineage, which was later joined by a return of so-called “second-generation” BA.2 sublineages (Figure 3), with BA.2.75.* and BA.2.3.20 being the most circulated. Since Summer 2022, each of those sublineages has amazingly converged with changes at the receptor-binding domain (RBD) residues R346, K444, L452, N450, N460, F486, F490, Q493, and S494 (see Supplementary Table S1) [27]. E484A remained instead stable, with 484K never detected, A484G seen only in BA.2.3.20, and A484T seen only in XBB.1.3. More recently, convergence in indels within the N-terminal domain (NTD), as previously recognized in Brazilian VOCs [28], was reported for Omicron sublineages: in particular, Y144del has been found in BA.4.6.3, BJ.1, BU.1, BQ.1.1.10, BQ.1.1.20, BQ.1.8.*, BQ.1.13.1, BQ.1.18, CR.1.3, and XBB.*) [29].

**Figure 4:**
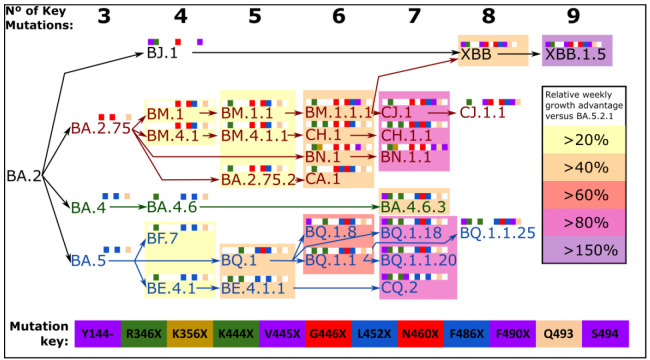
Step-wise accumulation of key Spike mutations involved in immune escape within SARS-CoV-2 Omicron sublineages increase the relative growth rate. Lineage name text is color coded, where BA.5 descendants are in blue text, BA.4 descendants in green text and BA.2.75 descendants are in red text. Each mutation is color coded as shown in the mutation key, and depicted as colored squares when present or white squares if absent. Number of key mutations of each lineage is summarized at the top. The F486P mutation is counted as two mutations due to the inherent increased fitness displayed by variants that carry this mutation relative to variants with F486S or F486V. Relative growth rates were calculated using BA.5 lineage as baseline, for groups of BA.4, BA.5, BA.2.75 and XBB descendant lineages with each exact total number of key mutations. Relative growth rates were calculated using global data, using CoV-Spectrum [30]. As of 26 December 2022.

This “variant soup” can be organized and stratified according to the number of key Spike mutations present, and although the number of key mutations acquired correlates well with increasing fitness, this is only so within each lineage, which shows that the biology of SARS-CoV-2 infection goes beyond what occurs in the Spike protein (Figure 4). At present, XBB.1.5 displays the highest relative growth advantage compared to the BA.5.2.1 baseline. Convergence was clearly observed at the amino acid level, with different nucleotide mutations leading to similar amino acid changes: e.g., N460K was caused by T22942A in BQ.1.*, XAW and some of the BA.5.2 sublineages, while it was caused by T22942G in BA.2.75.*(all lineages), BA.2.3.20, BS.1, BU.1, XBB, XAK and BW.1 (BA.5.6.2.1). Another impressive example of this convergent evolution is the Spike of BA.4.6.3, BQ.1.18 and BQ.1.1.20 independently acquiring the following amino acid changes since their last shared common ancestor: Y144del, R346T, and N460K. Additionally, BA.4.6.3 has acquired K444N, while BQ.1.18 and BQ.1.1.20 acquired K444T.

## 4. Escalating Immune Escape

**Figure 5:**
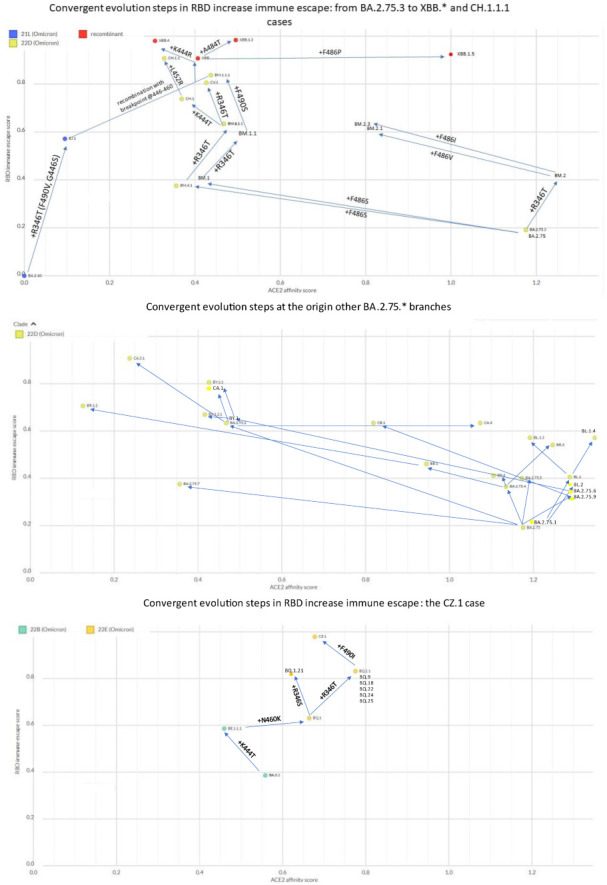
Evolutionary steps at the basis of the major Omicron branches (CZ.1, XBB.* and CH.1.1.1, and other BA.2.75.* descendants), showing progressive increases in RBD immune escape score (as calculated here: https://jbloomlab.github.io/SARS2_RBD_Ab_escape_maps/escape-calc/ (accessed on)). Chart created on NextStrain [31] (https://next.nextstrain.org/staging/nextclade/sars-cov-2/21L?gmin=15&l=scatter&scatterX=ace2_binding&scatterY=immune_escape&showBranchLabels=all (accessed on 26 December 2022).

SARS-CoV-2 evolution represents an accelerated movie of Darwinian selection. Variants that are more likely to escape vaccine- and infection-elicited immunity that are more fit expand at the expense of those less fit. While it may sound obvious, we now have formal evidence of such evolution, with PANGOLIN descendants invariably having increased RBD immune escape scores compared to parental strains (Figure 5). In this ongoing race, descendants invariably replace parents, as these are fitter in hosts with pre-existing immunity.

RBD immune escape can nowadays be estimated in silico based on in vitro data (https://jbloomlab.github.io/SARS2_RBD_Ab_escape_maps/escape-calc/ (accessed on 26 December 2022)). RBD immune escape is clearly a moving scale with an evolving asymptote. E.g., by changing vaccine composition [32] we are likely to reset the “game”.

## 5. ACE2 Affinity Fine Tuning

The binding rate of Spike protein for its receptor ACE2 is called affinity and can be estimated in silico (https://github.com/jbloomlab/SARS-CoV-2-RBD_DMS_Omicron/blob/main/results/final_variant_scores/final_variant_scores.csv (accessed on 26 December 2022). Several Omicron sublineages showed remarkable examples of further evolution at Spike residues that were already recently mutated. E.g.,

- BQ.1 already had K444T inherited from BE.1.1.1, but further mutated into 444M in the child BQ.1.1.17
- XBB.1 already had E484A inherited from the BA.2 parent, but further mutated into 484T in the child XBB.1.3
- BA.2.3 already had E484A inherited from the BA.2 parent, but further mutated into 484R in the child BA.2.3.20, which caused an impressive increase in ACE2 affinity (to whom K444R, L452M, and N460K contributed)
- BM.4.1.1 already had F486S inherited from the BM.4.1 parent but further mutated into 486P in CH.3
- BM.1.1.1 already had F486S inherited from the BM.1 parent but further mutated into 486P in the child CJ.1
- XBB.1 already had F486S inherited from the BM.1.1.1 parent but further mutated into 486P in the child XBB.1.5
- BA.2.75.2 already had F486S inherited from the BA.2.75 parent, but further mutated into 486L in the child CA.4
- BA.5.2.1 already had F486V inherited since BA.5, but further mutated to 486I in BF.12
- BW.1 already had F486V inherited from the BA.5 parent, but further mutated into 486S in the child BW.1.1

**Figure 6:**
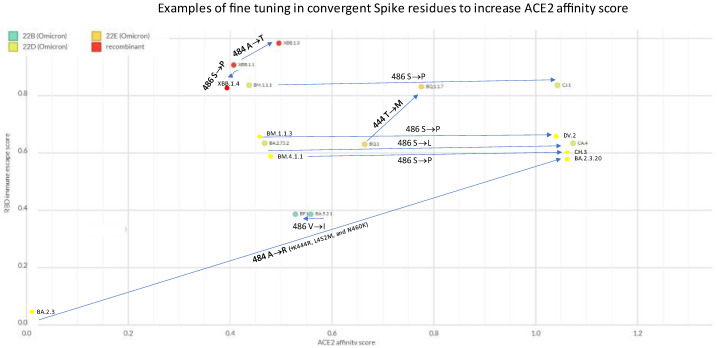
Sequential mutational events at the same Spike amino acid residues and change in ACE2 affinity score (as calculated here: https://github.com/jbloomlab/SARS-CoV-2-RBD_DMS_Omicron/blob/main/results/final_variant_scores/final_variant_scores.csv (accessed on 26 December 2022)). Chart created on NextStrain [31] (https://next.nextstrain.org/staging/nextclade/sars-cov-2/21L?gmin=15&l=scatter&scatterX=ace2_binding&scatterY=immune_escape&showBranchLabels=all (accessed on 26 December 2022)).

The majority of these examples manifest escalating affinities for ACE2, with the rest representing no change in ACE2 affinity (Figure 6). The F486S to S486P tuning (as well as V445A to A445P in XBB.*, or E484A to A484R in BA.2.3.20) represents a clear example of stepped 2-nucleotide changes, which typically happen when selective pressures are at work.

## 6. Mutually Exclusive Mutations

**Figure 7:**
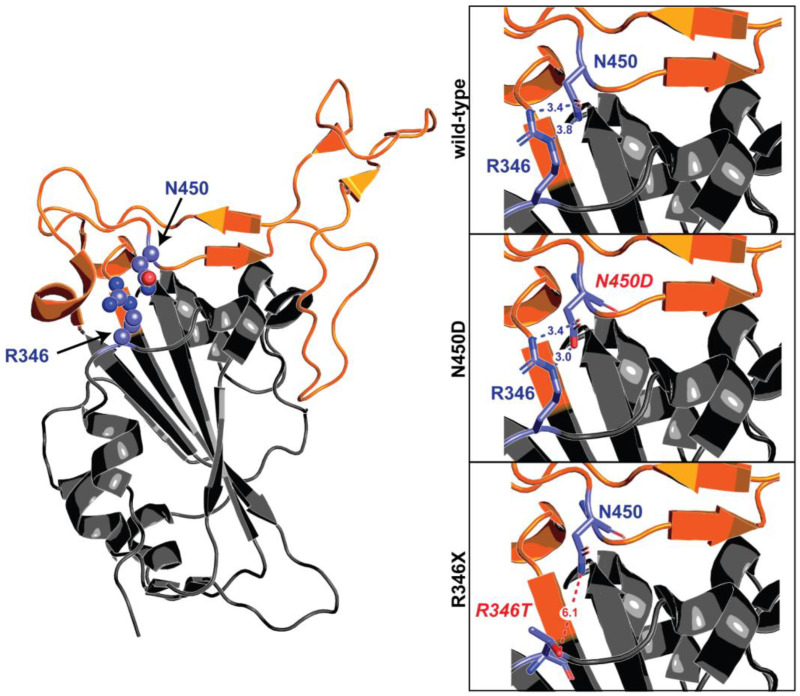
Mutually exclusive mutations at R346 and N450. The receptor binding domain of S is depicted in grey cartoon representation, with the receptor binding module (ACE2 interaction interface) highlighted in orange. Amino acids at the 346 and 450 positions are displayed as purple sticks. A zoomed-in view of the R346-N450 interaction in the ancestral domain, as well as the computationally modelled amino acid substitutions at those two positions, are portrayed in boxes to the right. In the wild-type sequence, the basic R346 sidechain interacts with the N450 residue through a pair of hydrogen bond interactions. N450D results in a similarly sized sidechain, but altered electrostatics. One hydrogen bond is maintained between the neutral oxygen of Asp and Nε of Arg, and a new salt bridge is formed between the anionic deprotonated oxygen of Asp and the cationic center of the guanidino group of Arg. In the case of R346X, any substitution except lysine would result in a side chain that is significantly shorter and non-cationic, thus dissolving the interactions between N450 or other common substitutions at that position.

Mutually exclusive mutations across the entire SARS-CoV-2 genome have been previously studied [33], but the vast constellation of Omicron sublineages provides an unique opportunity for an in-depth exploration of substitutions that are incompatible in combination. The best examples so far are N450X and R346X mutations, which have not yet been observed together in more than 6 millions of Omicron sequences. Two dipolar interactions exist between the carboxamide group of Asn and the guanidino group of Arg in the ancestral sequence, stabilizing the receptor binding module (RBM) tertiary fold (Figure 7, left). R346 resides within a short loop between helix α1 and beta strand 1. N450 is a constituent of the extended RBM insertion into the overall five-stranded antiparallel beta-sheet fold of the domain. As the RBM is the critical determinant for the interaction with ACE2, maintaining its optimal conformation through this stabilizing bond is likely to be essential for pathogenesis. N450D is a common substitution among Omicron lineages. This mutation would result in a similarly sized sidechain but different electrostatic properties (carboxamine → carboxylic acid). This substitution would likely result in a stronger interaction with position 450, as one H-bonding is maintained, and one is replaced with ionic salt bridge between the deprotonated oxygen and the basic guanidino group, provided that the residue at position 346 remains Arg. On the other hand, any substitution at position 346, with the exception of Lys, would result in a significantly shorter, non-cationic sidechain, which would abrogate this RBM-stabilizing interaction. R346K would partially maintain this interaction, replacing a bidentate linkage to N450 with a monodentate dipolar interaction. Thus, the observed mutual exclusivity of mutations at these two sites can be rationalized by their contributions to this stabilizing intradomain interaction.

Other combinations have been exceedingly rare so far, and seen only in cryptic lineages (e.g., F486P and K444 mutations), but no steric justifications can be found for them.

## 7. Epistasis

While the focus so far has been mostly on the Spike protein, it is likely that convergent evolution is acting on genes other than Spike. Given that the Spike protein is the best protective antigen for both infection and vaccines, mutations in other genes are more likely to provide fitness advantages if they affect Spike expression. E.g., ORF8 limits the amount of Spike proteins that reaches the cell surface and is incorporated into virions, reducing recognition by anti-SARS-CoV-2 antibodies [34]. ORF8 has accordingly been target of convergent evolution in Omicron (e.g., ORF8:S667F in BR.2.1, ORF8:G8x in XBB.1) and in SARS-related coronaviruses [35].

Other genes whose roles in Spike modulation are not clear are also converging, such as ORF1b:T1050, found in many BA.5.2.* sublineages, and XBE (T1050N) as well as XBC.* (T1050I).

## 8. Selective Pressures from Therapeutics Targeting the Spike Protein

There is a theoretical concern that, in addition to vaccines- and infection-elicited immunity, selective pressure by prophylactic and therapeutic anti-Spike monoclonal antibodies (mAb), can contribute to the emergence of novel SARS-CoV-2 sublineages [36]. A very few of those emerging sublineages could be fit enough to compete with the lineages that are dominating at that time to become locally or globally dominant.

While evolution can occur in the absence of selective pressures due to the intrinsic genomic MR (see section above), extended half-life mAbs (such as Evusheld™) administered for pre-exposure prophylaxis or therapy to chronically infected immunocompromised patients at subneutralizing concentrations provide ideal conditions to facilitate the emergence of mutants [37], for these patients often cannot clear the infection and have high viral loads. Establishing a cause-effect relationship is difficult, but intra-host evolution studies provide a highly suggestive temporal association [38]. mAbs have come of age since the advent of the SARS-CoV-2 Delta VOC, but because of the resistance of Omicron to most authorized mAbs, their use since Spring 2022 has been largely limited to Evusheld™ (for which cilgavimab was the only ingredient with residual activity) and bebtelovimab.

**Figure 8:**
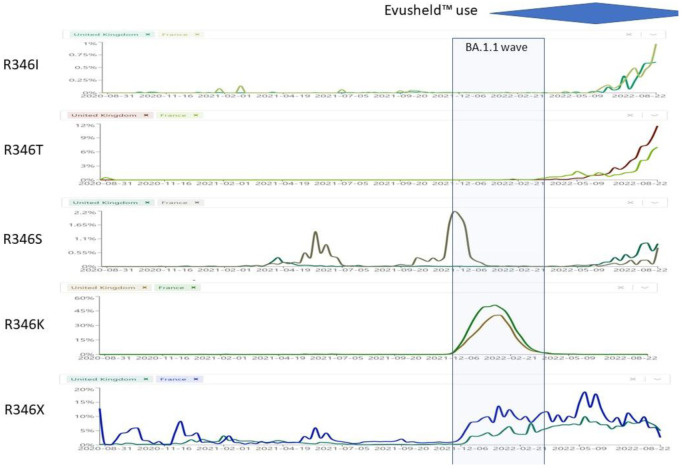
Prevalence of S:R346X mutations in the period 27 August 2022–13 September 2022 in UK versus France. Sourced from https://cov-spectrum.org (accessed on 26 November 2022). The blue area represents trends in Evusheld™ prescriptions in France and many other countries (but not UK).

We know from *in vitro* deep mutational scanning studies the exact mutations that cause resistance to each mAb. S:F486X mutations impart resistance to tixagevimab, S:R346X, S:K444X and S:S494X mutations impart resistance to cilgavimab, while S:K444X and S:V445X mutations impart resistance to bebtelovimab (Table 1). We wondered whether the recent increase in the circulation of Omicron sublineages with S:R346X mutations could partly be the result of selective pressure by mAbs. We compared the prevalence of R346X mutations in countries with high versus low usage of Evusheld™ (France vs. UK) or bebtelovimab (USA vs. UK) (Figure 8). UK also represents an ideal control because of its very high SARS-CoV-2 genome sequencing rate. We discuss these 2 scenarios in details below.

### 8.1. S:R346X

Different mutations can affect the R346 residue. R346G has been selected *in vitro* by cilgavimab + tixagevimab [39]. R346S occurred in vitro after 12 weeks of propagating SARS-CoV-2 in the presence of sotrovimab, and before the other epitope mutation (P337L) which leads to sotrovimab resistance [40]. R346I has been selected in vitro under the selective pressure from cilgavimab [41,42]. Lee et al. reported mutually exclusive substitutions at residues R346 (R346S and R346I) and E484 (E484K and E484A) of Spike protein and continuous turnover of these substitutions in 2 immunosuppressed patients [43]. Unfortunately, in vivo selection evidences are so far available for sotrovimab [44] but not for Evusheld™. It should be anyway noted that R346T [45,46] and R346I [47] have been reported to spontaneously develop and fix in 3 IC patients without any selective pressure.

While R346K was associated with the BA.1.1 wave (see Figure 8), the plethora of different Omicron sublineages that showed convergent evolution towards R346I, R346S or R346T is of concern.

- R346K (previously seen only in VOC Mu/B.1.621 [48]) occurred exclusively in BA.1.1, a sublineage that disappeared since May 2022, where it affected the interaction network in the BA.1.1 RBD/hACE2 interface through long-range alterations and contributes to the higher hACE2 affinity of the BA.1.1 RBD than the BA.1 RBD [49], and had increased resistance against Evusheld™ [50] and sotrovimab [51]. Only STI-9167 remained effective among the mAbs [52]. Beta + R346K, which was identified in the Philippines in August 2021, exhibited the highest resistance to 2 BNT61b2 doses-elicited sera among the tested VOCs [53]. After BA.1.1, R346K has not been detected worldwide in any sublineage.
- R346I occurs in more than 40 different Omicron sublineages, but it is most represented in BA.5.9 (38%), BA.4.1 (5%), BA.5.9 (4%), but also occurred in AY.39 (14%);
- R346S (previously seen only in a C.36.3 sublineage from Italy [54] (30.8%), occurs in more than 40 different Omicron sublineages but it is most represented in B.1.640.1 (18%), and in a few Delta sublineages (<2%)) occurs nowadays in BA.4.7 (13%), BA.5.2.1 (8.22%), BA.4 (2.8%).
- R346T occurs in more than 96 different Omicron sublineages, but it is mostly represented in BA.4.6 (44%), BA.5.2.1 (13%), BA.2 (8%), BA.2.74 (3%), BA.2.76 (12%), BA.4.1 (2.3%). In addition, it is a hallmark mutation of BA.1.23, BA.2.9.4, BL.1, BA.2.75.2, BA.2.80, BA.2.82, BA.4.1.8, BF.7 and BF.11. BA.4.6, BA.4.7, and BA.5.9 displayed higher humoral immunity evasion capability than BA.4/BA.5, causing 1.5 to 1.9-fold decrease in NT_50_ of the plasma from BA.1 and BA.2 breakthrough-infection convalescents compared to BA.4/BA.5. Importantly, plasma from BA.5 breakthrough-infection convalescents also exhibits significant neutralization activity decrease against BA.4.6, BA.4.7, and BA.5.9 than BA.4/BA.5, showing on average 2.4 to 2.6-fold decrease in NT_50_. R346S causes resistance to class 3 antibodies: bebtelovimab remains potent, while Evusheld™ is completely escaped by these subvariants [55].

### 8.2. S:K444X

The K444E/R mutations were reported in vitro after selection with cilgavimab [42]. Resistance studies with bebtelovimab selected the K444T escape mutations for BA.2 [56]. Ortega et al. found that K444R (previously found in the Beta VOC [57]), K444Q, and K444N mutations can change the virus binding affinity to the ACE2 receptor [58]. Weisblum et al. found that K444R/Q/N occurs after exposure to convalescent plasma [59]. Among largely diversified VOCs such as Delta, S:K444N was associated with reduced remdesivir binding and increased mortality [60].

## 9. Conclusions

The convergent evolution of Omicron sublineages appears to reflect the selective pressure exerted by previous infection- or vaccine-elicited immunity. Vaccines and perhaps antibody therapeutics have without doubt saved an untold number of lives but have the potential to modify the evolutionary trajectory of the virus. While other viruses such as influenza and HIV routinely produced new variants because of their mutagenicity, the scale at which SARS-CoV-2 has spun off new variants and lineages appears unprecedented in modern virology history. The SARS-CoV-2 vaccines reduce severe disease and mortality but do not confer sufficient immunity to prevent re-infection with viral replication in vaccinated hosts. Hence, we have the unusual situation of viral replication in hosts where the immune system is placing evolutionary pressure on the virus to select variants that can escape vaccine-elicited immunity in addition to infection-elicited immunity. Whether this rapid evolutionary trajectory is the result of viral replication properties, replication in immune hosts or both is unknown but conditions present in the past year of the pandemic have produced a remarkable natural experiment in viral evolution for which we cannot discern its conclusion.

Insights from structural biology has shown how some mutations are mutually exclusive, which could help the design of next-generation vaccines. However, the latter could reset the run for immune escape, perpetuating the never-ending game of host and pathogen. Viral recombination [61] (more than 50 lineages censed at the time of writing, with both simple and complex variants [62]) and sudden reemergence of former VOCs [63] have to be considered as further drivers for evolutionary saltation.

In this setting, polyclonal passive immunotherapies (such as plasma from convalescent and vaccinated donors [64,65]) appear more escape-resistant than monoclonal antibodies [66,67,68,69], and combo therapies should be urgently investigated and deployed in vulnerable populations, such as IC patients [70].

## Figures and Tables

**Table 1:** Heatmap of selected Spike RBD mutations in Omicron sublineages and their impact on authorized therapeutic anti-Spike mAbs. BAM: bamlanivimab; ETE: etesevimab; CAS: casirivimab; IMD: imdevimab; TIX: tixagevimab; CIL: cilgavimab; SOT: sotrovimab; BEB: bebtelovimab; REG: regdanvimab. Data sourced from the Stanford University Coronavirus and Antiviral Resistance Database (accessed online at https://covdb.stanford.edu/search-drdb (accessed on 30 November 2022)). Green means fold-reductions <5; Orange means fold-reduction 5–100; Red means fold-reduction in IC_50_ > 100 compared to wild-type; blank means no data available.

Spike Mutation		Main Lineages	BAM	ETE	CAS	IMD	CIL	TIX	SOT	BEB	REG
R346X	T	BA.2.3.22, BS.1.*, BP.1, DD.1, BJ.1, BL.1.*, BL.2.*, BL.5, BA.2.75.2.* (CA.*), BM.1.1.* (CJ.* and CV.*), BM.4.1.1.1.* (CH.*), BR.2.* and BR.3, BN.1, BA.2.75.6.* (BY.*), BA.2.75.9.* (CY.*), BA.2.76, BA.4.1.8 and BA.4.1.9, CS.1, BA.4.6.* (DC.*) and BA.4.7, BA.5.1.18 and BA.5.1.20, DE.2, BA.5.1.26.* (CU.*), BA.5.1.27 and BA.5.1.28, BF.7.*, BF.11.*, BA.5.2.6.* (CP.*), BA.5.2.13.* (CR.*), BA.5.2.25.* (DA.*), BA.5.2.39, BQ.1.1.* (CZ.*, CW.*, DK.*), BE.1.2.*, BE.1.4.2, BE.4.1.* (CQ.*), BE.5, BE.6, BE.7, BF.1, CK.1.2, CM.11, BL.6, XBB.*, XBD, XBE, XBF, XBG									
	E	BA.5.6.4									
	I	BF.33, CE.1									
	K	BA.1.1									
	R	BA.5.2.25, DB.2									
	S	BL.5, BF.13, BQ.1.21, BE.6									
K444X	M	CA.3.1, BR.1.*, BA.5.2.7, CY.1, BU.1, CG.1, BQ.1.17									
	N	BA.2.38.*, BA.4.6.3, BA.5.1.29, BV.2, BA.5.2.24, CK.* (DG.*), BE.4.2									
	R	BA.2.3.20.* (CM.*), CS.1, BF.16, BA.5.2.18, CR.1.*, CR.2, BA.5.2.41, CQ.1.*, XBB.4.*									
	T	CH.1.*, BR.4, BA.5.2.25, DB.1, DB.2, BA.5.2.36.* (CT.1), BE.1.1.1, BQ.1.* (CZ.*, CW.*, DK.*), BQ.2, BE.9, BA.5.2.46, BA.5.6.2.* (BW.1)									
V445	A	BA.4.6.2, BF.25, CP.1.1, BU.2, CR.1.2, BA.5.2.23, BE.1.2.1, BE.1.4.3, CQ.2									
	P	BJ.1, XBB.*									
G446	D	BA.5.2.30, CD.1									
	G	BR.4									
	S	BA.1.*, CM.8.*, BJ.1, BA.2.10.4, BH.1, BA.2.75.* (BL.*, CA.*, BM.*, CJ.*, CV.*, CH.*, BR.*, BN.*, BY.*, CB.*), BF.3.1, CP.1.3, CQ.1, XBB.*, XBC, XBD, XBF									
N450D		BU.3, CN.1, BA.5.2.32, BA.5.2.40, CC.1									
L452X	L	XBD									
	M	BP.1, BA.2.3.20.* (CM.*), XBC.1									
	Q	BH.1, BA.2.75.8									
	R	BS.1.*, CA.1, CA.3.1, CA.7, CV.1, CH.1.1, BA.2.75.4.* (BR.*), BY.1.1.*, BA.4.* (CS.*, DC.*), BA.5.* (BT.*, DH.*, DE.*, CU.*, CL.*, BF.*, BZ.*, CP.*, CY.*, BU.*, CR.*, BV.*, CN.*, CK.*, DG.*, DB.*, CG.*, CF.*, CD.*, CE.*, CT.*, DA.*, BE.*, BQ.*, CZ.*, CW.*, CC.*, CQ.*, BW.*, DK.*), XBE, XBG									
N460X	K	BS.1.*, BA.2.3.20.* (CM.*), DD.1, BA.2.75.* (BL.*, CA.*, BM.*, CJ.*, CV.*, CH.*, BR.*, BN.*, BY.*, CB.*), BA.4.6.3, CL.1, BF.33, CY.1, BU.1, CK.1, CK.2.*, DG.1, CK.3, DB.1, BQ.1.* (CZ.*, CW.*, DK.*), BE.4.2, BE.9, BW.1, BA.5.2.46, XBB.*, XBD, XBF									
	S	DC.1									
	Y	CP.3									
F486X	I	BM.2.3, BR.2.*, BF.7.12, BF.12									
	P	BA.2.10.4, CA.4, CJ.1, XBB.1.5, XBB.1.9.1, XBB.6.1, XBC.*, XBF, XBL									
	S	BA.2.75.2.* (CA.*), BM.1.* (CV.*), BM.4.1.* (CH.*), BR.1.2, BY.1.*, BA.2.75.7, CM.11, DS.1, XBB.*, XBD									
	V	BM.2.1, CB.1, BA.4.* (CS.*, DC.*), BA.5.* (BT.*, DH.*, DE.*, CU.*, CL.*, BF.*, BZ.*, CP.*, CY.*, BU.*, CR.*, BV.*, CN.*, CK.*, DG.*, DB.*, CG.*, CF.*, CD.*, CE.*, CT.*, DA.*, BE.*, BQ.*, CZ.*, CW.*, CC.*, CQ.*, BW.*, DK.*), XBE, XBG									
F490X	I	CZ.1									
	L	BL.1.3									
	S	BM.1.1.1.* (CJ.1), BN.1.*, BN.2.1., BN.3.1, BN.4, XBB.*, XBF									
	V	BJ.1, BL.1.4									
R493X	L	BA.2.3.21.1									
	Q	BA.2.10.4, BA.2.75.* (BL.*, CA.*, BM.*, CJ.*, CV.*, CH.*, BR.*, BN.*, BY.*, CB.*), BA.4.* (CS.*, DC.*), BA.5.* (BT.*, DH.*, DE.*, CU.*, CL.*, BF.*, BZ.*, CP.*, CY.*, BU.*, CR.*, BV.*, CN.*, CK.*, DG.*, DB.*, CG.*, CF.*, CD.*, CE.*, CT.*, DA.*, BE.*, BQ.*, CZ.*, CW.*, CC.*, CQ.*, BW.*, DK.*), XBB.*, XBC.*, XBD, XBE, XBF, XBG									
S494P		BA.2.10.4, CA.2, BN.1.*, BY.1.2.1, BQ.1.1.11, BQ.1.1.12, BQ.1.19									

## Data Availability

This manuscript generated no new datasets.

## Supplementary Materials

The following supporting information can be downloaded at: https://www.mdpi.com/article/10.3390/ijms24032264/s1.

## Abbreviations

IC: immunocompromised; MR: mutation rate; RBM: receptor-binding motif; VOC: variant of concern.
