# Supplementary material for: Convergent Evolution in SARS-CoV-2 Spike Creates a Variant Soup from Which New COVID-19 Waves Emerge
Source: Int J Mol Sci. 2023 Jan 23;24(3):2264. doi: 10.3390/ijms24032264 (PMC9917121; doi:10.3390/ijms24032264)
Supplement: Supplementary file 1 [file ijms-24-02264-s001.zip › ijms-2110643-supplementary.pdf]

Supplementary Table S1

Occurrence of selected amino acid mutations associated with immune escape within the Spike protein of SARS-CoV-2 in BA.2 and BA.4/5 Omicron sublineages. Modified from https://docs.google.com/spreadsheets/d/1OTWogpyvWNTlK0ww7TlDcI4l_SkZt3Z8nYiSp2YARys/edit?usp=sharing

**PANGO lineage**	nickname	**Y144del**	**R346**	**K356**	**K444**	**V445**	**G446**	**N450**	**L452**	**N460**	**F486**	**F490**	**R493**	**S494**
**BA.1**							S							
**BA.1.1**			K				S							
**BA.2**														
**BA.2.3**														
**BA.2.3.2**														
**BS.1**			T						R	K			Q	
BS.1.1			T	T					R	K			Q	
BS.1.2			T						R	K			Q	
**BA.2.3.16**														
BP.1			T						M					
**BA.2.3.20**	Basilisk				R			D	M	K			Q	
CM.1					R			D	M	K			Q	
CM.2					R			D	M	K			Q	
CM.3					R			D	M	K			Q	
CM.4					R			D	M	K			Q	
**CM.5**					R			D	M	K			Q	
CM.5.1					R			D	M	K			Q	
**CM.6**					R			D	M	K			Q	
CM.6.1					R			D	M	K			Q	
CM.7					R			D	M	K	S		Q	
**CM.8**					R		S	D	M	K			Q	
CM.8.1					R		S	D	M	K	S		Q	

CM.9					R			D	M	K			Q	
**BA.2.3.21**													Q	
DD.1			T							K			L	
**BA.2.10**														
**BA.2.10.1**														
BJ.1	Argus		T			P	S					V		
BA.2.10.4							S				P		Q	P
**BA.2.38**														
BA.2.38.1					N									
BA.2.38.2					N									
**BA.2.38.3**														
BH.1							S		Q					
BA.2.38.4					N									
**BA.2.75**	Centaurus						S			K			Q	
**BA.2.75.1**							S			K			Q	
**BL.1**			T				S			K			Q	
BL.1.1			T				S			K			Q	
BL.1.2			T				S			K			Q	
BL.1.3			T				S			K		L	Q	
BL.1.4			T				S			K		V	Q	
**BL.2**			T				S			K			Q	
BL.2.1			T				S			K			Q	
BL.3							S			K			Q	
BL.4							S			K			Q	
BL.5			S				S			K			Q	
**BA.2.75.2**	Chiron		T				S			K	S		Q	

CA.1			T				S		R	K	S		Q	
CA.2			T				S			K	S		Q	P
**CA.3**			T				S			K	S		Q	
CA.3.1			T		M		S		R	K	S		Q	
CA.4			T				S			K	P		Q	
CA.5			T				S			K	S		Q	
CA.6			T				S			K	S		Q	
CA.7			T				S		R	K	S		Q	
**BA.2.75.3**							S			K			Q	
**BM.1**							S			K	S		Q	
**BM.1.1**			T				S			K	S		Q	
**BM.1.1.1**	Mimas		T				S			K	S	S	Q	
CJ.1			T				S			K	P	S	Q	
BM.1.1.2			T				S			K	S		Q	
**BM.1.1.3**			T				S			K	S		Q	
CV.1			T				S		R	K	S		Q	
**BM.2**			T				S			K			Q	
BM.2.1			T				S			K	V		Q	
BM.2.2			T				S			K			Q	
BM.2.3			T				S			K	I		Q	
BM.3							S			K			Q	
**BM.4**							S			K			Q	
**BM.4.1**							S			K	S		Q	
**BM.4.1.1**			T				S			K	S		Q	
**CH.1**			T		T		S			K	S		Q	
CH.1.1			T		T		S		R	K	S		Q	
CH.2			T				S			K	S		Q	
BM.5							S			K			Q	
BM.6							S			K			Q	
**BA.2.75.4**							S		R	K			Q	

**BR.1**					M		S		R	K			Q	
BR.1.1					M		S		R	K			Q	
BR.1.2					M		S		R	K	S		Q	
**BR.2**			T				S		R	K	I		Q	
BR.2.1			T				S		R	K	I		Q	
BR.3			T				S		R	K			Q	
BR.4					T		G		R	K			Q	
**BA.2.75.5**				T			S			K			Q	
**BN.1**	Hydra		T	T			S			K		S	Q	
**BN.1.1**			T	T			S			K		S	Q	P
BN.1.1.1			T	T			S			K		S	Q	P
**BN.1.2**			T	T			S			K		S	Q	
BN.1.2.1			T	T			S			K		S	Q	
**BN.1.3**			T	T			S			K		S	Q	
BN.1.3.1			T	T			S			K		S	Q	
BN.1.4			T	T			S			K		S	Q	
BN.1.5			T	T			S			K		S	Q	
BN.1.6			T	T			S			K		S	Q	
BN.1.7			T	T			S			K		S	Q	
**BN.2**				T			S			K			Q	
BN.2.1				T			S			K		S	Q	
**BN.3**				T			S	D		K			Q	
BN.3.1				T			S	D		K		S	Q	
BN.4				T			S			K		S	Q	
BN.5				T			S			K			Q	
BN.6				T			S			K			Q	

**BA.2.75.6**	Dictys		T				S			K			Q	
**BY.1**			T				S			K	S		Q	
**BY.1.1**			T				S		R	K	S		Q	
BY.1.1.1			T				S		R	K	S		Q	
**BY.1.2**			T				S			K	S		Q	
BY.1.2.1			T				S			K	S		Q	P
BA.2.75.7							S			K	S		Q	
BA.2.75.8							S		Q	K			Q	
**BA.2.75.9**			T				S			K			Q	
CB.1			T				S			K	V		Q	
BA.2.75.10							S			K			Q	
BA.2.76			T											
**BA.4**									R		V		Q	
**BA.4.1**									R		V		Q	
BA.4.1.8			T						R		V		Q	
BA.4.1.9	Cetus		T						R		V		Q	
**BA.4.1.10**			T						R		V		Q	
CS.1			T		R				R		V		Q	
**BA.4.6**	Aeterna		T						R		V		Q	
BA.4.6.1			T						R		V		Q	
BA.4.6.2			T			A			R		V		Q	
BA.4.6.3			T		N				R	K	V		Q	
BA.4.6.4			T						R		V		Q	

**BA.4.6.5**			T						R		V		Q	
DC.1			T						R	S	V		Q	
BA.4.7			T						R		V		Q	
BA.4.8									R		V		Q	
**BA.5**									R		V		Q	
**BA.5.1**	Sphinx								R		V		Q	
BA.5.1.18			T						R		V		Q	
BA.5.1.19									R		V		Q	
BA.5.1.20			T						R		V		Q	
**BA.5.1.21**									R		V		Q	
BT.1									R		V		Q	
BT.2									R		V		Q	
**BA.5.1.22**									R		V		Q	
DH.1									R		V		Q	
**BA.5.1.23**									R		V		Q	
DE.1									R		V		Q	
DE.2			T						R		V		Q	
BA.5.1.24									R		V		Q	
BA.5.1.25									R		V		Q	
**BA.5.1.26**			T						R		V		Q	
CU.1			T						R		V		Q	
BA.5.1.27			T						R		V		Q	
BA.5.1.28			T						R		V		Q	
**BA.5.1.29**					N				R		V		Q	
CL.1									R	K	V		Q	
BA.5.1.30									R		V		Q	

**BA.5.2**	Triton								R		V		Q	
**BA.5.2.1**									R		V		Q	
**BF.1**									R		V		Q	
BF.1.1									R		V		Q	
BF.2									R		V		Q	
**BF.3**									R		V		Q	
BF.3.1							S		R		V		Q	
BF.4									R		V		Q	
BF.5									R		V		Q	
BF.6									R		V		Q	
**BF.7**	Minotaur		T						R		V		Q	
BF.7.1			T						R		V		Q	
BF.7.2			T						R		I		Q	
BF.7.3			T						R		V		Q	
**BF.7.4**			T						R		V		Q	
BF.7.4.1			T						R		V		Q	
BF.7.4.2			T						R		V		Q	
**BF.7.5**			T						R		V		Q	
BF.7.5.1			T						R		V		Q	
BF.7.6			T						R		V		Q	
BF.7.7			T						R		V		Q	
BF.7.8			T						R		V		Q	
BF.7.9			T						R		V		Q	
BF.7.10			T						R		V		Q	

BF.7.11			T						R		V		Q	
BF.7.12			T						R		V		Q	
**BF.7.13**			T						R		V		Q	
BF.7.13.1			T						R		V		Q	
BF.7.13.2			T						R		V		Q	
BF.8									R		V		Q	
BF.9									R		V		Q	
BF.10									R		V		Q	
**BF.11**	Python		T						R		V		Q	
BF.11.1			T						R		V		Q	
BF.11.2			T						R		V		Q	
BF.11.3			T						R		V		Q	
BF.11.4			T						R		V		Q	
BF.11.5			T						R		V		Q	
BF.12									R		I		Q	
BF.13			S						R		V		Q	
BF.14								D	R		V		Q	
BF.15									R		V		Q	
BF.16					R				R		V		Q	
BF.17									R		V		Q	
BF.18									R		V		Q	
BF.19									R		V		Q	
BF.20									R		V		Q	
BF.21									R		V		Q	
BF.22									R		V		Q	
BF.23									R		V		Q	
BF.24									R		V		Q	
BF.25						A			R		V		Q	
BF.26									R		V		Q	
BF.27									R		V		Q	
BF.28									R		V		Q	

BF.29									R		V		Q	
BF.30			T						R		V		Q	
**BF.31**									R		V		Q	
BF.31.1									R		V		Q	
BF.32								D	R		V		Q	
BF.33			I						R	K	V		Q	
BF.34			T						R		V		Q	
**BA.5.2.3**									R		V		Q	
BZ.1									R		V		Q	
**BA.5.2.6**			T						R		V		Q	
**CP.1**			T						R		V		Q	
CP.1.1			T			A			R		V		Q	
CP.1.2			T						R		V		Q	
CP.1.3			T				S		R		V		Q	
CP.2			T						R		V		Q	
CP.3			T						R	Y	V		Q	
CP.4			T						R		V		Q	
CP.5			T						R		V		Q	
CP.6			T						R		V		Q	
**BA.5.2.7**					M				R		V		Q	
CY.1					M				R	K	V		Q	
BA.5.2.13			T						R		V		Q	
**BA.5.2.16**									R		V		Q	
BU.1					M				R	K	V		Q	
BU.2						A			R		V		Q	
BU.3								D	R		V		Q	
**BA.5.2.18**					R				R		V		Q	
**CR.1**			T		R				R		V		Q	
CR.1.1			T		R				R		V		Q	
CR.1.2			T		R	A			R		V		Q	
CR.2					R				R		V		Q	

BA.5.2.19									R		V		Q	
**BA.5.2.20**									R		V		Q	
BV.1									R		V		Q	
BV.2					N				R		V		Q	
**BA.5.2.21**									R		V		Q	
CN.1								D	R		V		Q	
BA.5.2.22									R		V		Q	
BA.5.2.23						A			R		V		Q	
**BA.5.2.24**					N				R		V		Q	
CK.1					N				R	K	V		Q	
**CK.2**					N				R		V		Q	
**CK.2.1**					N				R	K	V		Q	
**CK.2.1.1**					N				R	K	V		Q	
DG.1					N				R	K	V		Q	
CK.3					N				R	K	V		Q	
**BA.5.2.25**			R		T				R		V		Q	
DB.1			T		T				R	K	V		Q	
DB.2			R		T				R		V		Q	
**BA.5.2.26**									R		V		Q	
CG.1					M				R		V		Q	
**BA.5.2.27**									R		V		Q	
CF.1									R		V		Q	
BA.5.2.28									R		V		Q	
BA.5.2.29									R		V		Q	
BA.5.2.30							D		R		V		Q	
**BA.5.2.31**									R		V		Q	
CD.1							D		R		V		Q	
CD.2									R		V		Q	

BA.5.2.32								D	R		V		Q	
**BA.5.2.33**									R		V		Q	
CE.1			I						R		V		Q	
BA.5.2.34			T						R		V		Q	
BA.5.2.35			T						R		V		Q	
**BA.5.2.36**					T				R		V		Q	
CT.1				T	T				R		V		Q	
BA.5.2.37									R		V		Q	
**BA.5.2.38**									R		V		Q	
DA.1			T						R		V		Q	
BA.5.2.39			T						R		V		Q	
BA.5.2.40								D	R		V		Q	
BA.5.2.41					R				R		V		Q	
**BA.5.3**									R		V		Q	
**BA.5.3.1**									R		V		Q	
**BE.1**									R		V		Q	
**BE.1.1**									R		V		Q	
**BE.1.1.1**					T				R		V		Q	
**BQ.1**	Typhon				T				R	K	V		Q	
**BQ.1.1**	Cerberus		T		T				R	K	V		Q	
**BQ.1.1.1**			T		T				R	K	V		Q	
CZ.1			T		T				R	K	V	I	Q	
BQ.1.1.2			T		T				R	K	V		Q	
BQ.1.1.3			T		T				R	K	V		Q	

BQ.1.1.4			T		T				R	K	V		Q	
BQ.1.1.5			T		T				R	K	V		Q	
BQ.1.1.6			T		T				R	K	V		Q	
BQ.1.1.7			T		T				R	K	V		Q	
BQ.1.1.8			T		T				R	K	V		Q	
BQ.1.1.9			T		T				R	K	V		Q	
BQ.1.1.10			T		T				R	K	V		Q	
BQ.1.1.11			T		T				R	K	V		Q	P
BQ.1.1.12			T		T				R	K	V		Q	P
BQ.1.1.13			T		T				R	K	V		Q	
**BQ.1.1.14**			T		T				R	K	V		Q	
CW.1			T		T		S		R	K	V		Q	
BQ.1.1.15			T		T				R	K	V		Q	
BQ.1.1.16			T		T				R	K	V		Q	
BQ.1.1.17			T		T				R	K	V		Q	
BQ.1.1.18			T		T				R	K	V		Q	
BQ.1.1.19			T		T				R	K	V		Q	
BQ.1.1.20			T		T				R	K	V		Q	
BQ.1.1.21			T		T				R	K	V		Q	
BQ.1.1.22			T		T				R	K	V		Q	
BQ.1.1.23			T		T				R	K	V		Q	
BQ.1.1.24			T		T				R	K	V		Q	

BQ.1.2					T				R	K	V		Q	
BQ.1.3					T				R	K	V		Q	
BQ.1.4					T				R	K	V		Q	
BQ.1.5					T				R	K	V		Q	
BQ.1.6					T				R	K	V		Q	
BQ.1.7					T				R	K	V		Q	
**BQ.1.8**					T				R	K	V		Q	
BQ.1.8.1					T				R	K	V		Q	
BQ.1.8.2					T				R	K	V		Q	
BQ.1.9			T		T				R	K	V		Q	
**BQ.1.10**					T				R	K	V		Q	
BQ.1.10.1					T				R	K	V		Q	
BQ.1.11					T				R	K	V		Q	
BQ.1.12					T				R	K	V		Q	
BQ.1.13					T				R	K	V		Q	
BQ.1.14					T				R	K	V		Q	
BQ.1.15					T				R	K	V		Q	
BQ.1.16					T				R	K	V		Q	
BQ.1.17					M				R	K	V		Q	
BQ.1.18			T		T				R	K	V		Q	
BQ.1.19					T				R	K	V		Q	P

BQ.1.20					T				R	K	V		Q	
BQ.1.21			S		T				R	K	V		Q	
BQ.1.22			T		T				R	K	V		Q	
BQ.1.23					T				R	K	V		Q	
BQ.1.24			T		T				R	K	V		Q	
BQ.1.25			T		T				R	K	V		Q	
BQ.1.26					T				R	K	V		Q	
BQ.2					T				R		V		Q	
**BE.1.1.2**									R		V		Q	
CC.1								D	R		V		Q	
**BE.1.2**			T						R		V		Q	
BE.1.2.1			T			A			R		V		Q	
BE.1.3									R		V		Q	
**BE.1.4**									R		V		Q	
BE.1.4.1									R		V		Q	
BE.1.4.2			T						R		V		Q	
BE.1.4.3						A			R		V		Q	
BE.1.4.4									R		V		Q	
BE.2									R		V		Q	
BE.3									R		V		Q	
**BE.4**									R		V		Q	
**BE.4.1**			T						R		V		Q	
**BE.4.1.1**			T		R				R		V		Q	
**CQ.1**			T		R				R		V		Q	

CQ.1.1			T		R				R		V		Q	
CQ.2			T		R	A			R		V		Q	
BE.4.2					N				R	K	V		Q	
BE.5			T						R		V		Q	
BE.6			S						R		V		Q	
BE.7			T						R		V		Q	
BE.8			T						R		V		Q	
BE.9					T				R	K	V		Q	
BA.5.3.5			T						R		V		Q	
**BA.5.5**									R		V		Q	
BA.5.5.3									R		V		Q	
**BA.5.6**									R		V		Q	
**BA.5.6.2**					T				R		V		Q	
BW.1					T				R	K	V		Q	
BA.5.6.3			T						R		V		Q	
BA.5.6.4			E						R		V		Q	
**BA.5.10**									R		V		Q	
**BA.5.10.1**			T						R		V		Q	
DF.1			T						R		V		Q	
BA.5.11			T						R		V		Q	
**XBB**	Gryphon		T			P	S			K	S	S	Q	
**XBB.1**			T			P	S			K	S	S	Q	
XBB.1.1			T			P	S			K	S	S	Q	
XBB.1.2			T			P	S			K	S	S	Q	
XBB.1.3			T			P	S			K	S	S	Q	

XBB. 1.4			T			P	S			K	S	S	Q	
XBB. 1.5			T			P	S			K	P	S	Q	
XBB. 2			T			P	S			K	S	S	Q	
**XBB. 3**			T			P	S			K	S	S	Q	
XBB. 3.1			T			P	S			K	S	S	Q	
**XBB. 4**			T		R	P	S			K	S	S	Q	
XBB. 4.1			T		R	P	S			K	S	S	Q	
XBB. 5			T			P	S			K	S	S	Q	
**XBC**							S				P		Q	
XBC. 1							S		M		P		Q	
XBC. 2							S				P		Q	
XBD			T				S		L	K	S		Q	
XBE			T						R		V		Q	
XBF			T				S			K	P	S	Q	
XBG			T						R		V		Q	
